# Prognostic value of non-alcoholic fatty liver disease for predicting cardiovascular events in patients with diabetes mellitus with suspected coronary artery disease: a prospective cohort study

**DOI:** 10.1186/s12933-020-01192-4

**Published:** 2021-01-07

**Authors:** Keishi Ichikawa, Toru Miyoshi, Kazuhiro Osawa, Takashi Miki, Hironobu Toda, Kentaro Ejiri, Masatoki Yoshida, Yusuke Nanba, Masashi Yoshida, Kazufumi Nakamura, Hiroshi Morita, Hiroshi Ito

**Affiliations:** 1 Department of Cardiovascular Medicine, Dentistry and Pharmaceutical Sciences, Okayama University Graduate School of Medicine, 2-5-1 Shikata-cho, Kita-ku, Okayama, 700-8558 Japan; 2 Department of Cardiovascular Medicine, Japanese Red Cross Okayama Hospital, Okayama, Japan; 3 Department of Cardiovascular Therapeutics, Dentistry, and Pharmaceutical Sciences, Okayama University Graduate School of Medicine, Okayama, Japan

**Keywords:** Cardiovascular disease, Computed tomography, Coronary artery calcium, Non-alcoholic fatty liver disease, Risk stratification

## Abstract

**Background:**

Risk stratification of cardiovascular events in patients with type 2 diabetes mellitus (T2DM) has not been established. Coronary artery calcium score (CACS) and non-alcoholic fatty liver disease (NAFLD) are independently associated with cardiovascular events in T2DM patients. This study examined the incremental prognostic value of NAFLD assessed by non-enhanced computed tomography (CT) in addition to CACS and Framingham risk score (FRS) for cardiovascular events in T2DM patients.

**Methods:**

This prospective pilot study included 529 T2DM outpatients with no history of cardiovascular disease who underwent CACS measurement because of suspected coronary artery disease. NAFLD was defined on CT images as a liver:spleen attenuation ratio < 1.0. Cardiovascular events were defined as cardiovascular death, nonfatal myocardial infarction, late coronary revascularization, nonfatal stroke, or hospitalization for heart failure.

**Results:**

Among 529 patients (61% men, mean age 65 years), NAFLD was identified in 143 (27%). Forty-four cardiovascular events were documented during a median follow-up of 4.4 years. In multivariate Cox regression analysis, NAFLD, CACS, and FRS were associated with cardiovascular events (hazard ratios and 95% confidence intervals 5.43, 2.82–10.44, p < 0.001; 1.56, 1.32–1.86, p < 0.001; 1.23, 1.08–1.39, p = 0.001, respectively). The global χ^2^ score for predicting cardiovascular events increased significantly from 27.0 to 49.7 by adding NAFLD to CACS and FRS (p < 0.001). The addition of NAFLD to a model including CACS and FRS significantly increased the C-statistic from 0.71 to 0.80 (p = 0.005). The net reclassification achieved by adding CACS and FRS was 0.551 (p < 0.001).

**Conclusions:**

NAFLD assessed by CT, in addition to CACS and FRS, could be useful for identifying T2DM patients at higher risk of cardiovascular events.

## Background

The prevalence of type 2 diabetes mellitus (T2DM) has been increasing rapidly worldwide [1]. T2DM is associated with a two- to four-fold increased risk of cardiovascular events compared with non-T2DM subjects [2, 3]. The prevention of cardiovascular events in T2DM patients has thus become a major concern. Although several clinical risk scores for predicting cardiovascular events have been proposed, there is currently no widely used risk stratification for T2DM patients. Previous studies showed that coronary artery calcium score (CACS) determined by coronary computed tomography (CT) provided additional information on cardiovascular events in T2DM patients beyond that provided by the commonly used Framingham risk score (FRS) [4, 5]. The latest American Heart Association and American College of Cardiology (AHA/ACC) guidelines for the primary prevention of atherosclerotic cardiovascular disease allowed the use of CACS in intermediate-risk patients if the risk level was uncertain [6]. Thus, CACS could be a useful factor for determining cardiovascular risk in patients with T2DM.

Growing evidence suggests that non-alcoholic fatty liver disease (NAFLD) is associated with cardiovascular events independently of established cardiovascular risk factors [7–9]. NAFLD is a frequent comorbidity of metabolic syndrome and T2DM [10]. Previous studies showed that metabolic syndrome and T2DM might be predictors of vascular damage [11, 12], and complex and bidirectional associations have been demonstrated between NAFLD and metabolic syndrome and T2DM [13]. We recently reported on the prognostic value of NAFLD assessed by non-enhanced CT in patients with suspected coronary artery disease who underwent coronary CT angiography [14], highlighting the benefits of concomitant assessment of liver fat content during the acquisition of coronary CT angiography to detect patients at higher risk of cardiovascular events.

We therefore hypothesized that the assessment of NAFLD using non-enhanced CT, in addition to CACS and FRS, might improve risk stratification for cardiovascular events in T2DM patients. We tested this hypothesis in a cohort of patients with suspected coronary artery disease who underwent CACS measurement, with the aim of evaluating the additional prognostic value of NAFLD compared with CACS and FRS in T2DM patients.

## Methods

### Study population and data collection

**Fig. 1:**
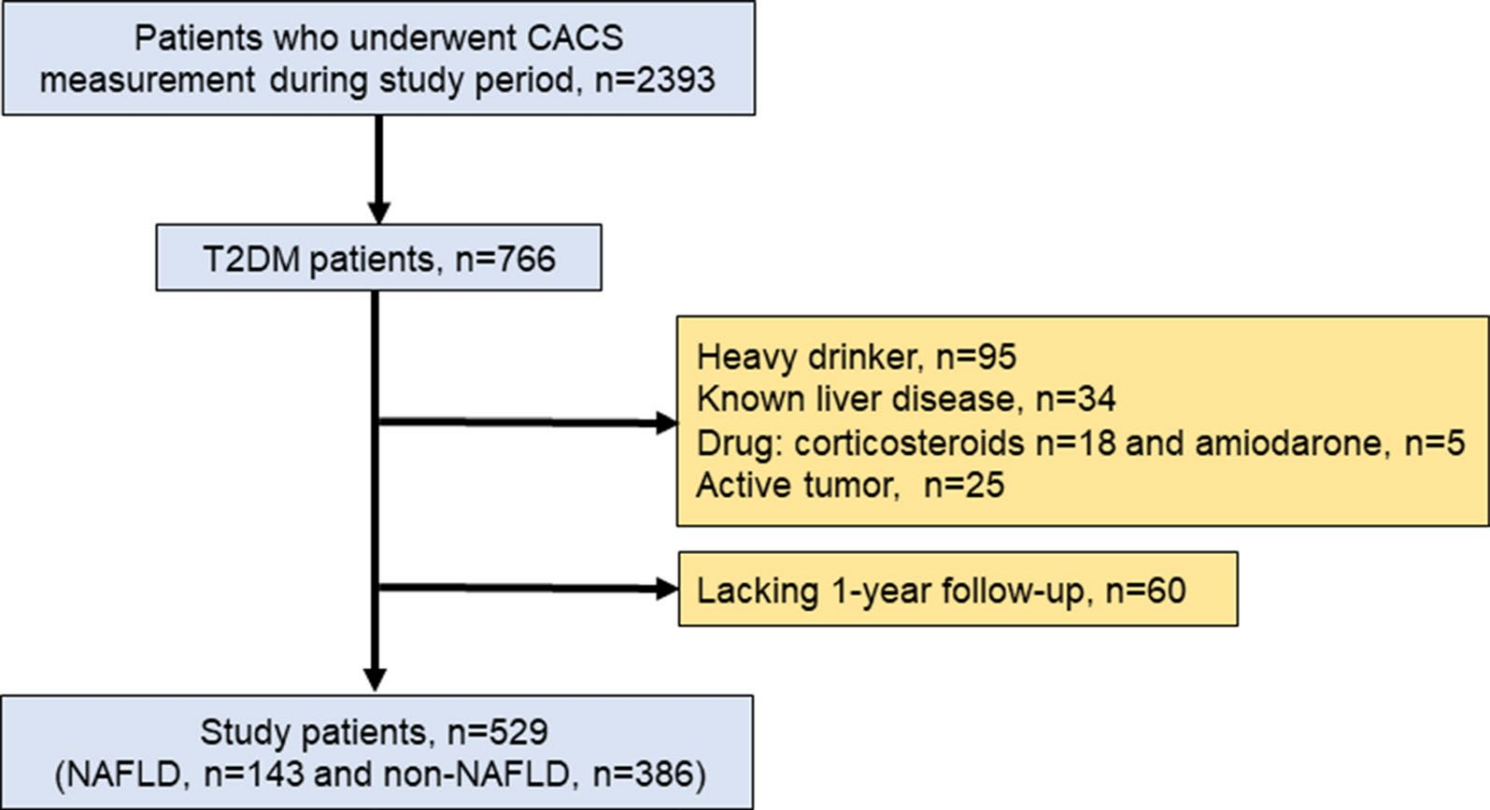
Flowchart of study design. *CACS* coronary artery calcium score, *T2DM* type 2 diabetes mellitus, *NAFLD* non-alcoholic fatty liver disease

Patient enrollment in this study is shown in Fig. 1. This prospective study enrolled 529 Japanese outpatients with T2DM from August 2011 to December 2016. The patients had no history of cardiovascular disease but had been referred to our hospital with suspected coronary artery disease. CACS was measured in 172 asymptomatic patients for risk stratification by evaluating coronary atherosclerosis, and in the remaining 357 patients with atypical symptoms [n = 44] or no symptoms [n = 313] because of abnormalities in rest or stress electrocardiograms. No patients had any history of cardiovascular diseases, including coronary artery disease, heart failure, or cerebrovascular disease. Patients were excluded if they consumed > 20 g of alcohol per day, had known liver disease, were using oral corticosteroids or amiodarone, or had a coexisting active tumor. All the patients underwent blood tests, measurement of CACS, and abdominal CT on the same day.

### Assessment of coronary calcification

CT imaging was performed using a Somatom Definition Flash scanner (Siemens Medical Solutions, Erlangen, Germany). CACS was measured using the following parameters: 120 kV, 150 mA, and 3-mm thickness. CACS was calculated using an automated computerized system (Virtual Place, Raijin; AZE Inc., Tokyo, Japan) and the Agatston method, which involved multiplying the area of each calcified plaque by a density factor determined by the peak pixel intensity within the plaque. The plaque-specific scores for all the slices were added together. The density factor was 1, 2, 3, or 4 for plaques with peak intensities of 130–199, 200–299, 300–399, and ≥ 400 Hounsfield units (HU), respectively [15]. In addition, patients were divided into three groups according to CACS: CACS 0, CACS (1–99), and CACS (≥ 100).

### Assessment of visceral adipose tissue and NAFLD

Abdominal non-contrast CT scans were carried out alongside cardiac CT, at a level including the liver, spleen, and umbilicus. The visceral adipose tissue area was assessed using a semi-automatic segmentation technique at the umbilical level [16]. Hepatic and splenic Hounsfield attenuations were measured using the maximum circular regions of interest in the liver and spleen (at least 1 cm^2^) [17]. Regions of interests in the liver were located at two segments (right anterior and right posterior) by avoiding the inclusion of large vessels or biliary structures [17]. The liver:spleen attenuation ratio was calculated using the mean HU measurement of the two right liver lobe regions of interest. In this study, we defined hepatic steatosis as a liver:spleen attenuation ratio < 1.0 [18]. NAFLD was finally diagnosed after other causes of hepatic steatosis were ruled out.

### Assessment of other risk factors

Hypertension was defined as a seated blood pressure > 140/90 mmHg or current treatment with antihypertensive medication. Dyslipidemia was defined as one or more of the following: serum triglycerides ≥ 150 mg/dL, high-density lipoprotein cholesterol < 40 mg/dL, low-density lipoprotein cholesterol ≥ 140 mg/dL, or current treatment with a lipid-lowering drug. Smoking status was defined as currently smoking or not smoking. Obesity was defined as a body mass index ≥ 25 kg/m^2^. FRS was calculated according to the algorithm presented by Wilson et al. to estimate the 10-year risk of a coronary heart disease event [19]. In addition, patients were classified into three groups according to the European Society of Cardiology recommendations as very-high, high, or moderate risk [20].

### Outcomes and follow-up

The patients were followed up prospectively from the date of CT. Clinical follow-up information was obtained by review of medical records or telephone interviews by attending physicians. The study endpoint was cardiovascular events defined as cardiovascular death, nonfatal myocardial infarction, late coronary revascularization, nonfatal stroke, or hospitalization due to heart failure. The diagnosis of myocardial infarction was based on the criteria of typical acute chest pain and persistent ST-segment elevation or positive cardiac enzymes. Late coronary revascularization was defined as percutaneous coronary intervention or coronary artery bypass grafting as indicated by the treating physician due to stable angina with a newly positive functional test. Patients with scheduled revascularization within 90 days of the CACS measurement were not counted as events and were censored at the time of first revascularization. Hospitalization for heart failure was defined as any unplanned stay overnight or longer in a hospital environment, for which heart failure was the principal reason for admission. Nonfatal stroke was defined as sudden-onset non-convulsive and focal neurological deficit persisting for > 24 h.

### Statistical analysis

Continuous variables were expressed as mean ± standard deviation or median with interquartile range. Dichotomous variables were expressed as number and percentage. Differences in continuous variables between two groups were analyzed by Student’s *t*-test or Mann–Whitney U-test, as appropriate. Categorical data were compared by χ^2^ tests. In subsequent analysis, triglyceride data were log-transformed because they did not show a normal distribution. Similarly, the distribution of Agatston score data was also highly skewed, and CACS was therefore log-transformed after adding 1 to all calcium scores to manage values of 0 (log[CACS + 1]). Kaplan–Meier curves were used to estimate cumulative rates of cardiovascular events. Differences between time-to-event curves were compared by log-rank tests. Annual event rates were calculated by dividing the 4-year Kaplan–Meier event rates by 4 and comparing them. The effects of variables on cardiovascular events were analyzed by Cox proportional hazard analysis, and the results were reported as hazard ratios (HR) with 95% confidence intervals (CI). The incremental value of NAFLD was assessed by global χ^2^ tests and receiver operating characteristic (ROC) curve analysis. C-statistics were calculated from the ROC curves and compared using the Delong test. The category-free net reclassification improvement was also calculated. All reported p values were two-sided and p < 0.05 was considered statistically significant. Statistical analyses were performed using SPSS statistical software (version 24; IBM Corp., Armonk, NY, USA) and the R statistical package (version 3.5.2; R Foundation for Statistical Computing, Vienna, Austria).

## Results

### Patient characteristics

The mean age of the study population was 65 years, 324 (61%) patients were men, and the median CACS was 63. Overall, 143 (27%) patients had CT evidence of NAFLD. The baseline characteristics of patients with and without NAFLD are shown in Table 1. Patients with NAFLD were younger (p < 0.001) and had a higher body mass index (p < 0.001) and visceral adipose tissue area (p < 0.001), and higher prevalences of dyslipidemia (p = 0.021) and obesity (p < 0.001) compared with patients without NAFLD. The proportion of very-high-risk patients was greater in patients with NAFLD than in patients without NAFLD (p = 0.003). Oral anti-hyperglycemic drugs were more frequent among patients with NAFLD. Patients with NAFLD also had higher levels of liver enzymes (p < 0.001) and triglycerides (p < 0.001), and lower levels of high-density lipoprotein cholesterol (p < 0.001). There was a significant difference in CACS between the two groups with higher CACS among non-NAFLD patients (p = 0.002). The mean dose-length product for abdominal CT was 251 mGy cm, and the effective dose for each imaging modality was 3.77 mSv, using a conversion coefficient of 0.015.

**Table 1:** Patient characteristics according to presence or absence of NAFLD

Variables	NAFLD, n = 143	No NAFLD, n = 386	p value
Age, years	60 ± 12	67 ± 12	< 0.001
Male gender	90 (63)	234 (61)	0.627
Body mass index, kg/m^2^	28 ± 4	24 ± 4	< 0.001
Visceral adipose tissue, cm^2^	139 ± 55	102 ± 57	< 0.001
Hypertension	108 (76)	265 (69)	0.124
Dyslipidemia	98 (69)	222 (58)	0.021
Current smoker	40 (28)	86 (22)	0.172
Obesity	99 (69)	126 (33)	< 0.001
β blocker	29 (20)	80 (21)	0.91
Calcium channel blocker	55 (39)	157 (41)	0.645
ACE-I or ARB	80 (56)	192 (50)	0.205
Statin	71 (50)	165 (43)	0.156
Insulin therapy	17 (12)	33 (9)	0.244
Oral antihyperglycemic drugs	94 (66)	198 (51)	0.003
Metformin	46 (32)	54 (14)	< 0.001
Thiazolidinediones	31 (22)	101 (26)	0.289
Alpha glucosidase inhibitor	20 (14)	62 (16)	0.558
DPP4 inhibitors	57 (40)	117 (30)	0.038
Creatinine, mg/dL	0.93 ± 0.91	1.14 ± 1.40	0.046
eGFR, mL/min/1.73 m^2^	72 ± 19	64 ± 23	< 0.001
AST, IU/L	29 ± 15	22 ± 13	< 0.001
ALT, IU/L	35 ± 22	22 ± 23	< 0.001
Total cholesterol, mg/dL	183 ± 37	183 ± 35	0.911
LDL cholesterol, mg/dL	108 ± 32	109 ± 30	0.767
HDL cholesterol, mg/dL	49 ± 16	56 ± 16	< 0.001
Triglyceride, mg/dL	150(102, 235)	109(81, 156)	< 0.001
HbA1c, %	7.6 ± 1.6	7.3 ± 1.7	0.076
CACS	30(0, 186)	107 (0, 536)	0.002
CACS category (0–99/≥100)	41/52/50	97/90/199	0.001
FRS	8.6 ± 3.9	8.6 ± 3.8	0.796
ESC very-high risk group	110 (77)	245 (64)	0.003

Data presented as mean ± standard deviation, number (%), or median [25th–75th percentile]

*NAFLD* non-alcoholic fatty liver disease, *ACE-I* angiotensin-converting enzyme inhibitors, *ARB* angiotensin receptor blockers, *eGFR* estimated glomerular filtration rate, *AST* aspartate aminotransferase, *ALT* alanine aminotransferase ratio, *LDL* low-density lipoprotein, *HDL-C* high-density lipoprotein, *HbA1c* glycated hemoglobin A1c, *CACS* coronary artery calcium score, *FRS* Framingham risk score, *ESC* European Society of Cardiology, *DPP4* dipeptidyl peptidase-4

### Outcome data

**Fig. 2:**
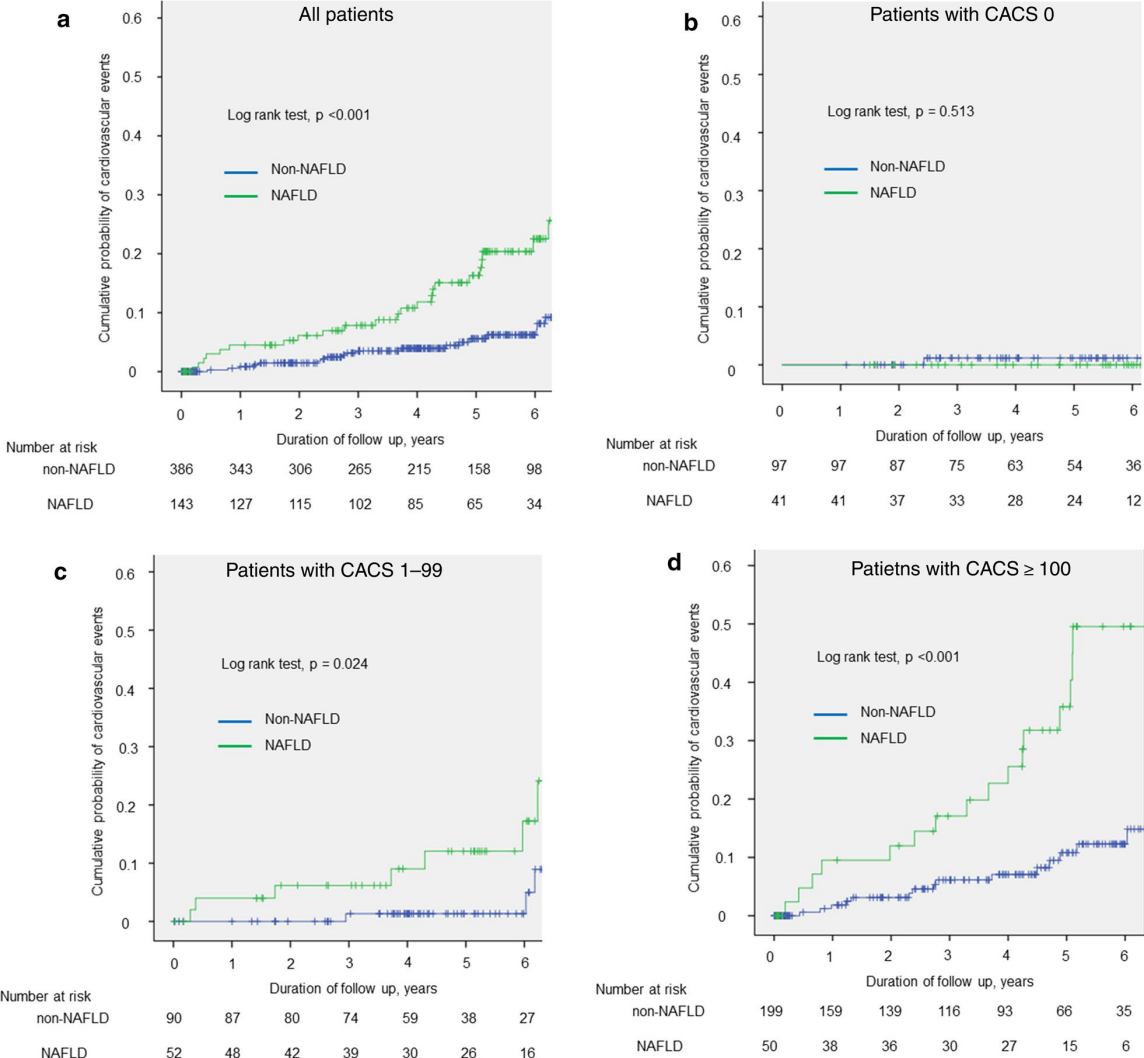
Kaplan–Meier curves showing cumulative incidence of cardiovascular events. Kaplan–Meier curves of cardiovascular events presented according to **a** presence or absence of NAFLD, and **b**–**d** presence or absence of NAFLD according to CACS categories. **a** All patietnts. **b** Patients wigh CACS 0. **c** Patients with CACS 1–99. **d** Patients with CACS ≥ 100. *CACS* coronary artery calcium score, *NAFLD* non-alcoholic fatty liver disease

Forty-six patients (11 NAFLD, 35 non-NAFLD) with scheduled revascularization within 90 days of the index CT were censored at the time of revascularization. Forty-four cardiovascular events were documented during a median follow-up of 4.4 years (23 events in NAFLD patients: 1 cardiovascular death, 5 stroke, 5 myocardial infarction, 10 late revascularization, 2 heart failure; 21 events in non-NAFLD patients: 3 stroke, 7 myocardial infarction, 8 late revascularization, 3 heart failure). Kaplan–Meier curves showed the cumulative event-free survivals for cardiovascular events in patients stratified by CACS (0, 1–100 or ≥ 100), with or without NAFLD. The annual cardiovascular event rate in patients with NAFLD was significantly higher than that in patients without NAFLD (2.95% vs. 0.98%; p < 0.001) (Fig. 2a). In addition, patients were divided into three groups according CACS and compared in relation to the presence or absence of NAFLD (Fig. 2b–d). Patients with NAFLD and CACS ≥ 100 had a significantly higher incidence of cardiovascular events. The annual rates of cardiovascular events in patients with CACS 0 with and without NAFLD were very low and similar in both groups (0.00% vs. 0.30%; p = 0.513) (Fig. 2b). The annual rates of cardiovascular events in patients with CACS 1–99 and ≥ 100 were significantly higher in NAFLD compared with non-NAFLD patients (2.25% vs. 0.33%; p = 0.024, Fig. 2c; and 6.40% vs. 1.78%; p < 0.001, Fig. 2d, respectively). As shown in Table 2, univariate Cox regression analysis identified presence of NAFLD, CACS, and FRS as factors associated with cardiovascular events. Furthermore, multivariate Cox regression analysis identified the presence of NAFLD, CACS, and FRS as associated with cardiovascular events (HR, 95% CI 5.43, 2.82–10.44, p < 0.001; 1.56, 1.32–1.86, p < 0.001; 1.23, 1.08–1.39, p = 0.001, respectively).

**Table 2:** Univariate and multivariate predictors of cardiovascular events

Variables	Univariate		Multivariate	
	Hazard ratio (95% CI)	p value	Hazard ratio (95% CI)	p value
Age	1.04 (1.01–1.07)	0.010	1.00 (0.96–1.04)	0.938
Male, gender	1.64 (0.87–3.10)	0.127	2.50 (1.01–6.18)	0.047
Hypertension	1.28 (0.63–2.60)	0.496	0.77 (0.38–1.57)	0.467
Dyslipidemia	1.28 (0.69–2.36)	0.439	1.14 (0.59–2.22)	0.695
Current smoker	1.21 (0.61–2.39)	0.592	0.75 (0.35–1.62)	0.467
Obesity	1.12 (0.62–2.03)	0.704	0.96 (0.49–1.88)	0.912
β blocker	0.56 (0.22–1.43)	0.229		
Calcium channel blocker	1.46 (0.82–2.67)	0.189		
ACE-I or ARB	1.20 (0.66–2.17)	0.554		
Statin	1.05 (0.58–1.90)	0.872		
Insulin therapy	0.87 (0.44–1.73)	0.690		
Oral antihyperglycemic drugs	0.81 (0.45–1.47)	0.488		
Metformin	0.36 (0.13–1.00)	0.050		
Thiazolidinediones	1.95 (0.90–4.19)	0.089		
Alpha glucosidase inhibitor	0.50 (0.18–1.40)	0.189		
DPP4 inhibitors	1.33 (0.72–2.45)	0.368		
Visceral adipose tissue (> 100 cm^2^)	1.91 (1.01–3.60)	0.046		
NAFLD	2.91 (1.61–5.25)	< 0.001	5.43 (2.82–10.44)	< 0.001
Total cholesterol	1.00 (0.99–1.01)	0.934		
LDL cholesterol	1.00 (0.99–1.01)	0.944		
HDL cholesterol	0.99 (0.96–1.01)	0.151		
Log (triglyceride)	1.48 (0.84–2.60)	0.178		
HbA1c	0.93 (0.77–1.12)	0.424		
Log (CACS + 1)	1.48 (1.27–1.72)	< 0.001	1.56 (1.32–1.86)	< 0.001
FRS	1.14 (1.05–1.24)	0.002	1.23 (1.08–1.39)	0.001
ESC very-high risk group	1.41 (0.72–2.73)	0.316		

*ACE-I* angiotensin-converting enzyme inhibitors, *ARB* angiotensin receptor blockers, *NAFLD* non-alcoholic fatty liver disease, *LDL* low-density lipoprotein, *HDL-C* high-density lipoprotein, *HbA1c* glycated hemoglobin A1c, *CACS* coronary artery calcium score, *FRS* Framingham risk score, *ESC* European Society of Cardiology, *DPP4* dipeptidyl peptidase-4

### Comparison of predictive performances for cardiovascular events

**Fig. 3:**
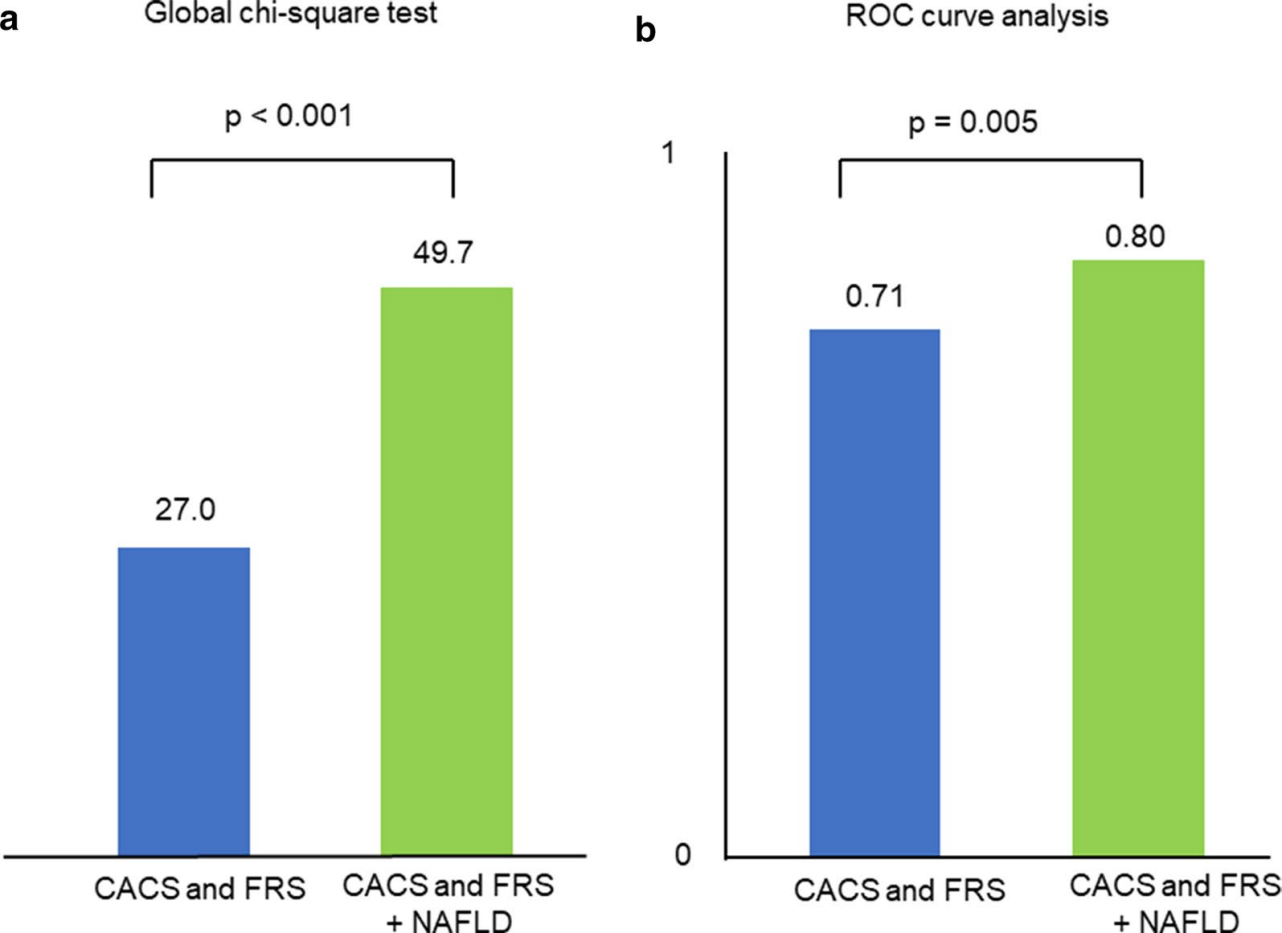
Incremental prognostic value of NAFLD in addition to CACS and FRS. **a** Global χ^2^ test. **b** Receiver operating characteristic curve analysis. *NAFLD* non-alcoholic fatty liver disease, *CACS* coronary artery calcium score, *FRS* Framingham risk score

We investigated the incremental value of adding NAFLD compared with CACS and FRS alone for predicting cardiovascular events. The global χ^2^ score and ROC curve analysis were calculated to assess the incremental predictive value of NAFLD. Adding NAFLD to log (CACS + 1) and FRS significantly increased the global χ^2^ score from 27.0 to 49.7 (p < 0.001) (Fig. 3a). The results of ROC analysis comparing the area under the curve for each group are shown in Fig. 3b. Adding NAFLD significantly increased the C-statistic of Model 1 (FRS + log [CACS + 1]) from 0.71 to 0.80 (p = 0.005). The net reclassification index achieved by adding log (CACS + 1) and FRS was 0.551 (p < 0.001). The addition of NAFLD to CACS and FRS thus improved the predictability of cardiovascular events.

## Discussion

This study demonstrated that the presence of NAFLD in non-enhanced CT images, in addition to CACS and FRS, improved the risk classification of cardiovascular events in T2DM patients. However, this study was conducted in a cohort of T2DM patients with suspected coronary artery disease, and further studies are needed to determine if the results apply to all T2DM patients.

Several lines of evidence have shown that NAFLD is associated with an increased risk of cardiovascular events in T2DM patients. Lee et al. showed that NAFLD was independently associated with progression of carotid intima-media thickness, as a well-established surrogate marker of subclinical atherosclerosis, in T2DM patients [21]. In an observational study of 2103 T2DM patients, NAFLD was associated with an increased incidence of cardiovascular events after adjustment for multiple risk factors (HR 1.96, 95% CI 1.4–2.7) [22]. However, the mechanisms by which NAFLD increases the risk of cardiovascular events are complex and not fully understood. A previous study showed that the histological severity of NAFLD was associated with increased arterial stiffness and endothelial dysfunction [23]. In addition, inflammatory cytokines increased in line with the severity of liver disease in NAFLD patients [24]. The presence of systemic inflammation promoted by cytokines secreted from the liver leads to endothelial dysfunction, altering vascular tone and enhancing vascular plaque formation. This mechanism was supported by a clinical study that found a significant association between the severity of NAFLD and both surrogate markers of atherosclerosis and an increased risk of cardiovascular mortality in NAFLD patients [25–28].

CACS is a well-established surrogate marker of subclinical coronary artery atherosclerotic plaque burden, which can predict risk beyond the established cardiovascular risk score. Budoff et al. reported that CACS was independently and strongly associated with cardiovascular events, and CACS > 100 signified at least a 7.5% 10-year risk of cardiovascular events regardless of age, sex, or ethnicity among 6814 subjects from the general population [29]. CACS is also used to predict cardiovascular risk in T2DM patients, with elevated CACS in T2DM compared with non-T2DM subjects [30]. The Diabetes Heart Study comprising 1123 T2DM patients demonstrated that CACS predicted cardiovascular events more accurately than FRS [4]. In addition, the 2019 AHA/ACC guidelines for the primary prevention of atherosclerotic cardiovascular disease included the measurement of CACS for patients in intermediate-risk groups [6]. These data support the possible use of CACS as a means of assessing risk for cardiovascular outcomes in T2DM patients.

Hepatic steatosis has been reportedly associated with the presence of coronary artery calcium in some studies [31–33]. In addition, Sung et al. reported that hepatic steatosis was independently associated with coronary artery calcium progression [34]. However, the association between NAFLD and CACS has been inconsistent across studies, especially in T2DM patients. In a study of 213 participants with T2DM, NAFLD was not associated with CACS in patients with glycated hemoglobin A1c (HbA1c) < 7%, but was significantly associated with CACS in patients with HbA1c ≥ 7% [35]. In contrast, McKimme et al. reported no significant association between hepatic steatosis and CACS in T2DM patients [36]. Kim et al. reported an association between NAFLD and the prevalence of CACS, but this association was attenuated and was no longer significant after adjusting for insulin resistance [37]. The current study also found no association between NAFLD and higher CACS. Given that our results indicated that NAFLD and CACS were independent factors, the combination of NAFLD and CACS might improve the identification of T2DM patients at higher risk of cardiovascular events.

Ultrasonography is commonly used to assess liver fat infiltration in clinical practice; however, non-enhanced CT can also be useful for diagnosing liver fat. Previous studies showed that a liver:spleen ratio < 1.0 could be used effectively to diagnose the presence of liver fat with high reproducibility [18, 38, 39]. However, the prevalence of NAFLD in T2DM in this study was lower than that reported in other studies [40], in which NAFLD was mostly diagnosed by ultrasonography and magnetic resonance imaging. On the other hand however, our study applied CT, which was reported to have a lower sensitivity for diagnosing hepatic steatosis compared with ultrasonography and magnetic resonance imaging, especially in cases with mild steatosis (< 30% steatosis) [41]. The present study may thus have included patients with moderate to severe hepatic steatosis.

NAFLD is closely associated with obesity [22], and the prevalence of NAFLD has been reported to increase in parallel with increasing severity of obesity [42]. Recently, severe obesity among children and adolescents has recently become a significant public health concern [43]. Furthermore, pediatric fatty liver disease clustered with cardiometabolic risk factors, associated with an increase in subsequent adult cardiovascular mortality among adolescents with severe obesity [44]. A healthier diet and physical activity should thus be promoted among adolescents with obesity to mitigate the cardiometabolic risk.

This study had several limitations that need to be addressed. First, the number of patients and cardiovascular events were relatively small. In addition, our study population only consisted of Japanese patients with suspected coronary artery disease, and the results therefore cannot be applied directly to the T2DM population or to other ethnic groups. Second, the histological severity of liver damage was not confirmed in this study. However, CT is a useful tool for diagnosing NAFLD, without the complications associated with invasive methods. The association between CACS and histologic findings of NAFLD, such as ballooning grade, need to be evaluated in future studies. Third, we have no data about the duration of T2DM, which has been reported to increase the risk of cardiovascular events [45]. Fourth, longitudinal information on changes in medications, risk factor control, and changes in body mass index and lifestyle during the follow-up period was not available. Finally, FRS was originally developed in Western societies and is therefore not accurate in Asians. The Suita score has been proposed and validated as an alternative score for predicting coronary heart disease in Japanese populations [46]. The application of the Suita score instead of FRS did not affect the findings of this study (data not shown).

## Conclusions

This study demonstrated the potential incremental prognostic value of NAFLD assessed by non-enhanced CT, in addition to CACS, for risk stratification of cardiovascular events in T2DM patients with suspected coronary artery disease. Further studies are needed to validate the applicability of NAFLD and CACS examination by non-enhanced CT to all T2DM patients.

## Data Availability

The datasets used and/or analyzed during the current study are available from the corresponding author on reasonable request.

## Abbreviations

CACS: Coronary artery calcium score
CT: Computed tomography
FRS: Framingham risk score
HbA1c: Glycated hemoglobin A1c
HU: Hounsfield unit
NAFLD: Non-alcoholic fatty liver disease
ROC: Receiver operating characteristic
T2DM: Type 2 diabetes mellitus
